# Influenza A virus hemagglutinin mutations associated with use of neuraminidase inhibitors correlate with decreased inhibition by anti-influenza antibodies

**DOI:** 10.1186/s12985-019-1258-x

**Published:** 2019-11-29

**Authors:** Natalia A. Ilyushina, Takashi E. Komatsu, William L. Ince, Eric F. Donaldson, Nicolette Lee, Julian J. O’Rear, Raymond P. Donnelly

**Affiliations:** 1 Division of Biotechnology Review and Research II, Food and Drug Administration CDER, WO Bldg. 52/72, Room 2105, 10903 New Hampshire Avenue, Silver Spring, MD 20993 USA; 2 Division of Antiviral Products, Food and Drug Administration, 10903 New Hampshire Avenue, Silver Spring, MD 20993 USA

**Keywords:** Hemagglutinin (HA), Neuraminidase (NA), Neuraminidase inhibitors (NAI), Influenza A virus, Antiviral resistance

## Abstract

**Background:**

Vaccination and the use of neuraminidase inhibitors (NAIs) are currently the front lines of defense against seasonal influenza. The activity of influenza vaccines and antivirals drugs such as the NAIs can be affected by mutations in the influenza hemagglutinin (HA) protein. Numerous HA substitutions have been identified in nonclinical NAI resistance-selection experiments as well as in clinical specimens from NAI treatment or surveillance studies. These mutations are listed in the prescribing information (package inserts) for FDA-approved NAIs, including oseltamivir, zanamivir, and peramivir.

**Methods:**

NAI treatment-emergent H1 HA mutations were mapped onto the H1N1 HA1 trimeric crystal structure and most of them localized to the HA antigenic sites predicted to be important for anti-influenza immunity. Recombinant A/California/04/09 (H1N1)-like viruses carrying HA V152I, G155E, S162 N, S183P, and D222G mutations were generated. We then evaluated the impact of these mutations on the immune reactivity and replication potential of the recombinant viruses in a human respiratory epithelial cell line, Calu− 3.

**Results:**

We found that the G155E and D222G mutations significantly increased viral titers ~ 13-fold compared to the wild-type virus. The hemagglutination and microneutralization activity of goat and ferret antisera, monoclonal antibodies, and human serum samples raised against pandemic A(H1N1)pdm09 viruses was ~ 100-fold lower against mutants carrying G155E or D222G compared to the wild-type virus.

**Conclusions:**

Although the mechanism by which HA mutations emerge during NAI treatment is uncertain, some NAI treatment-emergent HA mutations correlate with decreased immunity to influenza virus.

## Background

Influenza virus continues to have a major impact on global health and is responsible for millions of cases of respiratory illness and hundreds of thousands of hospitalizations annually in the United States alone [1]. The envelope glycoproteins, hemagglutinin (HA) and neuraminidase (NA), mediate host cell attachment and release, respectively, and are the primary targets of the protective antibody-mediated immune response. HA has functionally defined immunodominant antigenic sites that primarily map to the globular domain of the glycoprotein and surround the receptor binding site (RBS) [2]. Circulating influenza viruses gradually accumulate HA mutations, primarily in the antigenic sites targeted by neutralizing antibodies, and these changes frequently allow escape from the antibody-mediated memory immune response. This process is known as antigenic “drift” and is likely driven by selection imposed by prevailing immunity in the host population, resulting in the need to periodically update the vaccine strains. Influenza virus can escape the antibody response through substitutions that induce conformational changes in the antigenic sites (epitopes), thus limiting antibody binding. Moreover, the modulation of viral HA receptor binding avidity can also lead to antigenic change and escape from antibody neutralization [3, 4].

Many of the antiviral drug products that are either FDA-approved or in development for prophylaxis or treatment of influenza virus infection target the HA and/or NA glycoproteins and they include NA inhibitors (NAIs), monoclonal antibodies (mAbs), and vaccines. The activity of these drugs and vaccines may be affected by changes in the dynamic HA and NA molecules selected by the clinical use of these therapeutic agents. For example, influenza viruses with amino acid substitutions and/or deletions associated with reduced susceptibility to NAIs have been identified in cell culture selection studies, NAI-treated patients, as well as in circulating viruses from untreated individuals [5–11]. Genetic analysis showed that reduced susceptibility to NAIs is associated with mutations in the viral NA and/or HA proteins and many of these mutations are listed in the NAI package inserts [12–14]. Although the mechanistic basis for NAI treatment-emergent mutations in HA has yet to be defined, it is likely that their predicted effect of lowering receptor binding avidity compensates for reduced NA activity [5–11]. The link between HA antibody escape and occurrence of compensatory NA mutations that result in acquisition of increased NAI resistance has been documented [15]. However, it is not clear if HA mutations associated with clinical use of NAIs correlate with decreased immune reactivity to anti-influenza antibodies. The present study demonstrates that NAI treatment-emergent HA mutations can result in altered antigenic profiles and may potentially impact antibody-mediated virus inhibition.

## Methods

### Generation of recombinant viruses

Eight plasmids of the 8 gene segments of wild-type A/California/04/09 A(H1N1)pdm09 (CA/04) virus were kindly provided by Dr. Robert G. Webster at St. Jude Children’s Research Hospital, Memphis, TN. Recombinant viruses were generated by DNA transfection of 293 T cells, and the point mutations were inserted into the HA gene of wild-type virus using a Quickchange site-directed mutagenesis kit (Stratagene) [16]. Stock viruses were prepared in Madin-Darby canine kidney (MDCK) cells at 37 °C for 72 h and their entire HA and NA genes were sequenced to verify the presence of the desired HA1 mutations and the absence of any additional HA/NA substitutions. The recombinant viruses were designated according to their HA1 substitutions. All experimental work was performed in a biosafety level 2 laboratory approved for use with these strains by the U.S. Department of Agriculture and the U.S. Centers for Disease Control and Prevention.

### Mapping of NAI treatment-emergent H1 HA mutations and antigenic sites

**Fig. 1:**
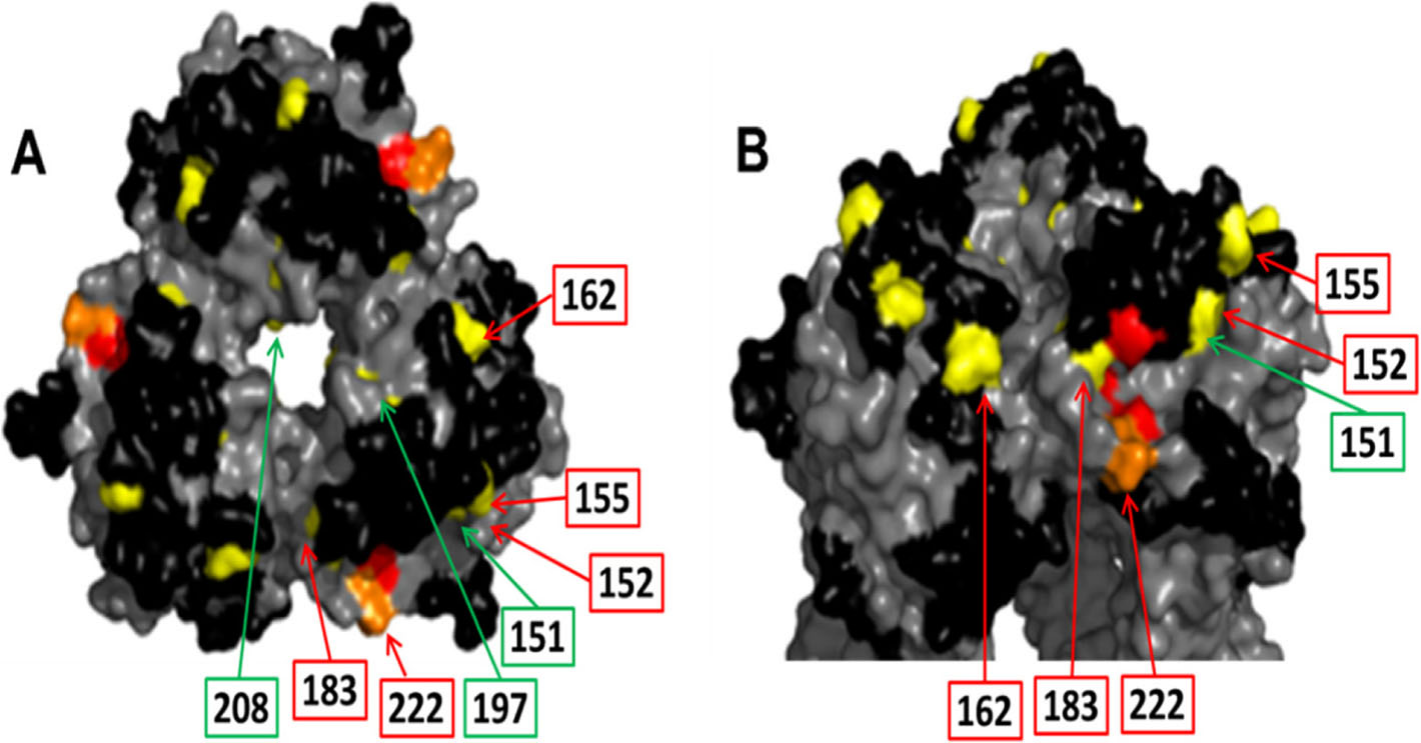
Mapping of NAI-treatment emergent HA mutations on the H1N1 HA1 trimer. The NAI-treatment emergent HA mutations emerged in the H1N1 HA1 domain (listed in Table 1) were mapped onto the H1 HA1 trimer crystal structure (PDB:3LZG) to determine if these substitutions occurred in previously identified H1N1 antigenic sites [17]. The trimeric HA structure is shown in gray (•). Epitopes described in the literature for H1N1 were colored black (•). NAI-treatment emergent HA1 mutations are colored yellow (•). Amino acids associated with the RBS are shown in red (•) and NAI-treatment emergent substitutions occurred within the RBS are shown in orange (•). Panel A, H1N1 top view and Panel B, H1N1 side view, rotated 60 degrees upward. Labelling of amino acid positions was limited to one monomer. Boxes and lines colored in red indicate substitutions that were selected for evaluation in the HI and MN assays. Boxes and lines colored in green indicate substitutions that were not evaluated in the HI and MN assays

Structural bioinformatics was used to determine if the NAI treatment-emergent HA1 mutations can be mapped to previously described H1N1 HA antigenic sites. The X-ray crystal structure of the CA/04 HA protein (PDB:3LZG) was downloaded from the protein databank and analyzed using MacPymol (DeLano Scientific LLC). This X-ray crystal structure was selected for our analysis because it is the same structure for which A(H1N1) epitopes were mapped previously [17]. The putative antigenic sites that are conserved between influenza A(H1N1) viruses and A(H1N1)pdm09 were identified based on comparison of their HA amino acid sequences and structures (Fig. 1). Each of the antigenic sites was mapped onto the HA1 trimer and colored black to distinguish these sites from the rest of the structure, which is colored in gray. NAI treatment-emergent HA1 mutations identified in cell culture or in clinical studies were mapped onto the structure using yellow or orange (for substitutions occurred in the RBS).

### Hemagglutination inhibition (HI) and microneutralization (MN) assays

A hemagglutination inhibition (HI) assay was performed with 0.5% chicken red blood cells by a standard method [26]. We used goat and ferret antisera as well as human convalescent sera from individuals who were confirmed to be infected with A(H1N1)pdm09 virus and a panel of 4 mAbs to HA of the CA/04 strain. Human sera were collected from anonymous donors (i.e., Donor 1, Donor 2, and Donor 3) in the licensed BioLife plasma collection centers during 2009–2010 and these serum samples were commercially obtained from Baxter Inc. Four antibodies to CA/04 HA, mAbs 28665, 28666, 28667, and 28668, were obtained through BEI Resources, NIAID, NIH. Virus-neutralizing titers were determined by infection of MDCK cells and expressed as the reciprocal of the highest serum dilution that neutralized 50% of fifty 50% tissue culture infectious doses (TCID_50_) of virus after incubation at 37 °C for 72 h.

### Viral replication kinetics in the presence or absence of NA inhibitor

To determine multistep growth curves for each virus in the presence or absence of oseltamivir carboxylate (5 nM = 5 effective concentration-50 for the CA/04 virus [27]), human lung epithelial Calu− 3 cells were inoculated via the apical side with the H1N1 viruses at a multiplicity of infection of 0.001 plaque-forming units (PFU)/cell. After incubation for 1 h, the cells were washed and overlaid with medium with or without 5 nM oseltamivir carboxylate, 0.3% bovine serum albumin, and 1 μg/ml l-(tosylamido-2-phenyl)ethylchloromethylketone-treated trypsin. The supernatants were collected at 24, 48, and 72 h post-infection and stored at − 70 °C until titration.

### Statistical analysis

Virus yields were compared by analysis of variance (ANOVA) with Dunnett’s multiple comparisons test. A probability value of 0.05 was prospectively chosen to indicate that the findings were not the result of chance alone.

## Results

### NAI treatment-emergent H1 HA mutations listed in NAI package inserts

A group of HA substitutions that were identified in nonclinical NAI resistance-selection experiments or in clinical specimens from NAI treatment or surveillance studies were analyzed in this study (Table 1). Many of these mutations are described in the current FDA-approved NAI package inserts [12–14]. In general, NAI-resistant substitutions are included in drug product labels based on clinical observation, cell culture selection, and phenotype data. HA substitutions are generally included as NAI treatment-emergent mutations if they meet one of the following criteria: (i) selected in cell culture in the presence of NAI; (ii) observed as treatment-emergent in more than one patient, (iii) observed as treatment-emergent in a single patient at positions identified in cell culture as impacting drug susceptibility; and (iv) observed in surveillance or baseline clinical study samples at positions identified in cell culture as impacting susceptibility. It is worth noting that HA/NA substitutions observed at baseline in clinical studies or in surveillance samples are included in the approved drug product labels because there are examples of circulating amino acid polymorphisms that clearly reduce susceptibility of influenza virus to antivirals. Examples include NA H275Y in the pre-2009-pandemic H1N1 lineage and M2 S31 N in the majority of currently circulating seasonal influenza viruses [28] as well as other viruses [29, 30]. However, it is likely that the impact of HA1 mutations on susceptibility to NAIs is highly strain- and target tissue-dependent. Therefore, some polymorphisms may not be selected by or alter susceptibility to NAIs in the currently circulating strains.

**Table 1:** NAI treatment-emergent H1 HA mutations included in current FDA-approved NAI package inserts

H1 HA mutation included in NAI package inserts^a^	HA mutations selected in cell culture/associated with reduced susceptibility to NAI in cell culture		Strains containing corresponding treatment-emergent HA mutation	Strains from untreated patients containing corresponding HA mutation (or HA mutation at the same residue)	Corresponding NAI package insert
	HA mutation (if different from listed) and/or stain, in which the HA mutation was observed	NA changes observed together with corresponding HA mutation			
D125S^b^ (129)	A/Puerto Rico/8/34 (H1N1) [14]	**–**	**–**	N125D, A(H1N1)pdm09 [18, 19]	RAPIVAB® [14]
L151P (154)	H151Q, A/Wuhan/259/95 (H1N1) [20]	E119V	A(H1N1)pdm09 [21]	**–**	RELENZA® [13]
V152I (155)	T152A, NWS/G70C (H1N9) [6]^c,d^	–	–	A(H1N1)pdm09 [18, 21, 22]	RELENZA® [13]
G155E (158)	A(H1N1)pdm09 [10]^c,d^	N146S	**–**	**–**	RELENZA® [13]
S162 N^e^ (165)	NWS/G70C (H1N9) [7]^c,d^	–	A(H1N1)pdm09 [23]	A(H1N1)pdm09 [21, 22, 24]	RELENZA® [13]
S183P (186)	S183F, NWS/G70C (H1N9) [7]^c,d^	E119G	**–**	A(H1N1)pdm09 [18, 23]	RELENZA® [13]
A197T (200)	A/WSN/33 (H1N1) [9]^d^	Deletion 92–362	**–**	A(H1N1)pdm09 [23]	RELENZA® [13]
R208K (211)	A/Puerto Rico/8/34 (H1N1) [14]	**–**	**–**	**–**	RAPIVAB® [14]
D222G (225)	A/Hokkaido/15/02 (H1N1) [11]^c^	Y155H, V114I	–	A(H1N1)pdm09 [18, 21–23]	RELENZA® [13]

“ –” - not identified

^a^HA1 mutations were identified in a variety of strains and reported using different numbering systems. Numbering in this table is subtype-specific and based on corresponding positions in A/California/04/2009 (H1N1) as described previously [25]. Numbering begins after the predicted signal peptide. H3 HA numbering is shown in parenthesis

^b^Substitution could not be reliably mapped to the HA structure due to ambiguous HA numbering coordinates (the system of numbering could not be unambiguously determined based on the available information or did not match the expected wild-type amino acid in the reported strain)

^c^HA mutation independently reduced susceptibility to NAIs in cell culture

^d^Drug-dependent phenotype was demonstrated in cell culture

^e^HA1 mutation introduces potential N-linked glycosylation site

### Mapping of NAI treatment-emergent HA mutations

We mapped the amino acid substitutions located in the HA1 domain of A(H1N1) and/or A(H1N1)pdm09 viruses onto the H1 HA1 trimeric crystal structure (Fig. 1). These mutations were identified in nonclinical NAI resistance-selection experiments or clinical specimens from treatment or surveillance studies and are listed in the corresponding NAI drug package inserts (Table 1). We also visualized the HA structural association with previously identified antigenic sites [17]. Most of the NAI treatment-emergent H1 HA1 mutations mapped to the antigenic sites predicted to be important for immunity. These mutations included D125S, G155E, S162 N, and D222G (H1 numbering convention is used here and throughout the text). Substitutions at positions S183 and D222 were associated with the RBS and have been shown to impact escape from neutralizing antibodies [3, 31] (Fig. 1a, b). Substitution R208K mapped to the interior of the trimer below the surface-exposed globular domain distal to the HA1 antigenic sites.

### Effect of NAI treatment-emergent HA mutations on viral growth in the presence or absence of NA inhibitor

**Fig. 2:**
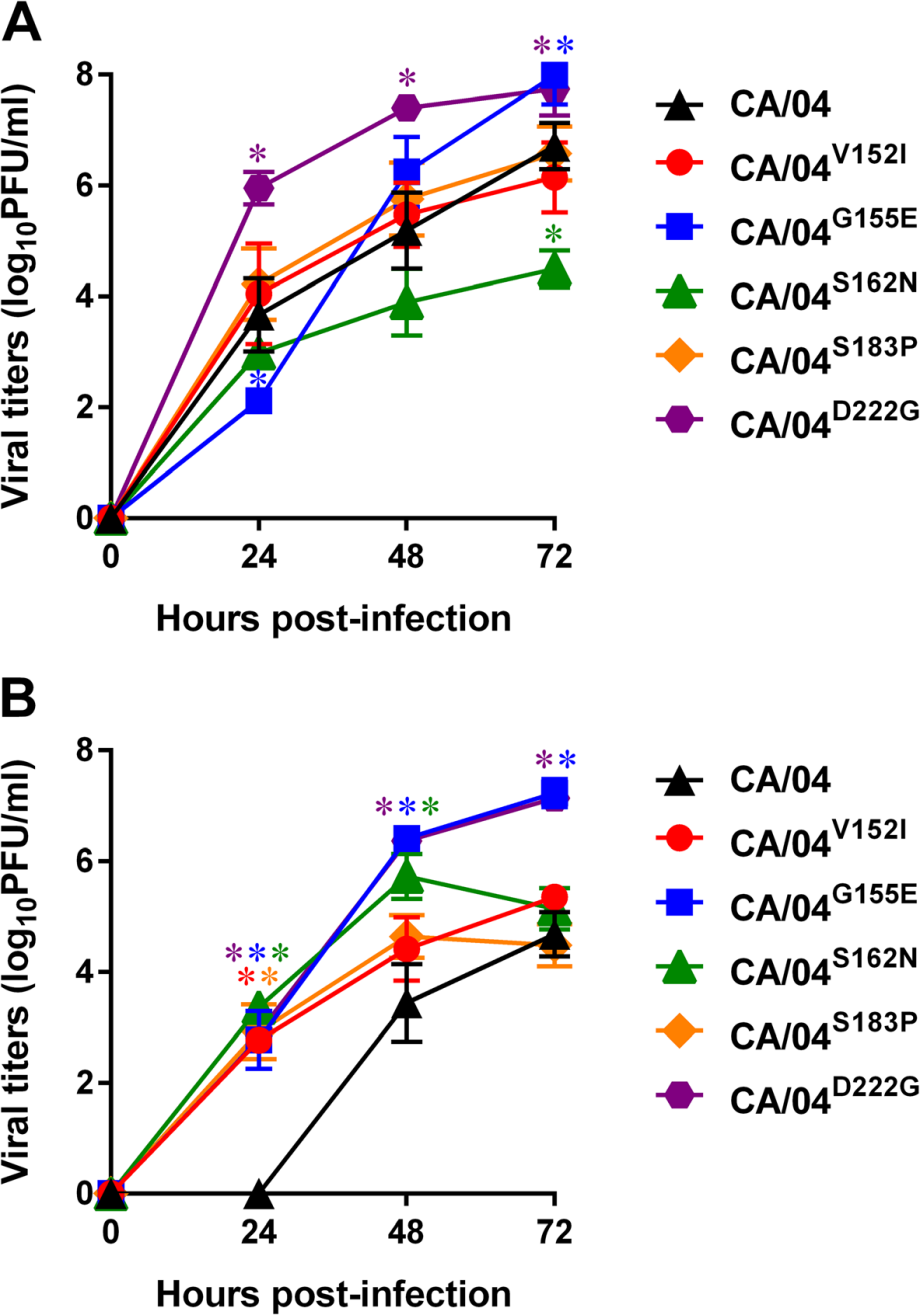
Replication of wild-type and mutant H1N1 influenza A viruses in the absence (**a**) and presence of 5 nM oseltamivir carboxylate (**b**) in Calu-3 cells. The results are expressed as log_10_PFU/ml from three to four independent experiments performed on different days. *P < 0.05, compared to the values for the wild-type CA/04 virus (one-way ANOVA)

We evaluated the replicative ability of the recombinant H1N1 mutants by assaying their virus yields in comparison to those of the parental virus after multiple replication cycles in Calu− 3 cells. As shown in Fig. 2a, the CA/04^G155E^ and CA/04^D222G^ viruses grew to significantly higher titers than the wild-type virus at 72 h post-infection (~ 1.1 log_10_PFU/ml, *P* < 0.05). Both mutants also formed larger plaques than the parental CA/04 virus in MDCK cells (*P* < 0.05, data not shown). To determine if any of the NAI treatment-emergent HA1 mutations are associated with a drug-dependent phenotype, we examined the replication of the H1N1 mutants in the presence of 5 nM oseltamivir carboxylate in Calu− 3 cells (Fig. 2b). We observed that all of the HA1 mutations could rescue the weak growth capacity of the wild-type virus, thus masking any replication defect and increasing NAI resistance (~ 2.8-fold increase in viral titers compared to CA/04, *P* < 0.05).

### Effect of NAI treatment-emergent HA mutations on antibody reactivity

To evaluate the impact of NAI treatment-emergent HA1 mutations on immune reactivity, we assessed the sensitivity of viruses containing selected HA1 substitutions to several anti-H1 mAbs and polyclonal antisera in HI and MN assays. HI of goat and ferret antisera raised against pandemic A(H1N1)pdm09 viruses was significantly reduced (~ 20-fold↓) against recombinant CA/04^G155E^ and CA/04^D222G^ viruses carrying the G155E and D222G substitutions, respectively, compared to the wild-type CA/04 virus (Table 2). The HI activity of mAbs 28665 and 28668, which target antigenic site Sa [32], was reduced > 48-fold. The S183P substitution resulted in ~ 4-fold drop in 28665 titers and ~ 2-fold drop in 28666 and 28667 titers. There were no significant changes in ferret antisera or mAb HI activity against V152I and S162 N. To further characterize the antigenic properties of the mutant viruses, we performed MN assays using mAbs and human serum samples against CA/04. Consistent with the HI results, the MN activity of mAbs 28665 and 28668 and human serum samples collected from three different donors was reduced by >170-fold against recombinant CA/04^G155E^ and CA/04^D222G^ compared to CA/04 (Table 3). Overall, our results indicate that NAI treatment-emergent HA1 mutations may alter the antigenic profiles of A(H1N1)pdm09 and result in decreased antigenicity of the HA protein. However, this effect is selective because not all substitutions resulted in demonstrable changes in the HI and MN assays.

**Table 2:** Antigenic characterization of wild-type and mutant H1N1 influenza A viruses by HI assay

H1N1 virus	Titer ^a^							
	Goat antiserum against CA/04	Ferret antiserum against:			mAbs against CA/04:			
		CA/04	A/CA/07/09	A/TN/1–560/09	28665	28666	28667	28668
CA/04^V152I^	0	0	0	0	0	0	0	0
CA/04^G155E^	-3	-3	-3	-3	>-5	1	1	>-6
CA/04^S162N^	1	0	1	0	0	1	−1	0
CA/04^S183P^	0	0	0	0	-2	−1	-1	0
CA/04^D222G^	−2	− 6	−3	− 4	>-5	0	0	>-6

^a^Values represent the differences between the HI titers of antisera or mAbs (reciprocals of the serum/antibody dilutions that inhibited 8 hemagglutination units of virus) in the reactions with the wild-type CA/04 and mutant H1N1 viruses in log_2_ units

**Table 3:** Antigenic characterization of wild-type and mutant H1N1 influenza A viruses by MN assay

H1N1 virus	mAbs against CA/04:				Human sera against CA/04:		
	28665	28666	28667	28668	Donor 1	Donor 2	Donor 3
CA/04^V152I^	3^a^	0	2	−1	0	−1	0
CA/04^G155E^	−6	0	1	−9	−6	− 9	− 8
CA/04^S162N^	4	0	5	−1	0	0	0
CA/04^S183P^	0	0	−1	− 1	0	− 1	0
CA/04^D222G^	−4	0	3	−8	−4	−4	−5

^a^Values represent the differences between the neutralizing mAb titers (reciprocals of antibody dilutions that neutralized 50 TCID_50_s of virus) in the reactions with the wild-type CA/04 and mutant H1N1 viruses in log_2_ units

## Discussion

The relationship between antigenic drift and concomitant changes in HA receptor binding specificity/avidity has been well documented [3, 4, 33–35]. Due to the proximity of the antigenic sites to the RBS, antigenic changes selected by neutralizing antibodies are often accompanied by changes in HA receptor binding properties [3, 33, 36]. Immune pressure elicited by infection and/or immunization may favor influenza HA substitutions that facilitate antibody escape both by altering antigenicity and by increasing HA avidity for cell surface receptors. Subsequent absence of immune pressure may further induce compensatory substitutions that reduce avidity often by increasing the negative charge of the HA region and alter antigenicity [3, 33, 34]. On the other hand, HA mutations that alter viral receptor affinity/specificity can contribute to NAI resistance by allowing efficient virus release from infected cells without the need for significant NA activity [5, 37]. Although the effect of NAI treatment-emergent HA mutations on NAI susceptibility in humans remains uncertain, they are usually listed in the package inserts for NAI drug products. The possibility exists that NAIs may be associated with development of HA mutations that permit escape from natural or vaccine immunity by changing HA receptor avidity. The selective pressures mediated by NAIs and acquired immunity may also work together to accelerate the selection of beneficial compensatory HA mutations affecting receptor specificity, antigenicity, and/or functional compatibility with the NA protein.

Indeed, most of the NAI treatment-emergent HA1 mutations studied here involved changes in receptor binding avidity and specificity and, therefore, demonstrated drug-dependent phenotype in Calu-3 cells. The G155E mutation, which maps to the antigenic site Sa, and the S183P mutation, which is located within the RBS and overlaps with antigenic site Sb [36, 38], have been shown to significantly increase HA receptor binding to α2,6-linked sialyl receptors [38]. According to the previous studies, the S183P mutation enhanced virulence by altering binding to sialyl receptors in a mouse animal model [3, 35, 37]. Moreover, the high frequency of the S183P mutation in contemporary H1N1 viruses in 2017–2018 (~ 28%) indicates that this mutation is being strongly selected for in humans. The D222G mutation, which maps to the antigenic site Ca [17, 31, 39], was shown to be closely associated with the enhanced virulence of A(H1N1)pdm09 virus through the increased binding affinity to α2,3-linked sialyl receptors, while maintaining α2,6 specificity [40–43]. In addition, S183P and D222G altered receptor binding avidity of the A/Puerto Rico/8/34 (H1N1) strain; however, whether a specific mutation results in increased or decreased receptor binding avidity depends on its genetic context [3, 31, 34].

There is considerable uncertainty regarding the impact of HA mutations both on NAI susceptibility in in vitro systems and on virus inhibition and clinical response to NAIs in vivo. As noted in several NAI drug product labels, the impact of HA mutations on antiviral activity of NAIs is not well characterized in humans and is likely to be influenza virus strain-dependent. While some of the HA mutations within the RBS have been associated with a drug-dependent phenotype [5, 7], the NAI-reduced susceptibility phenotype associated with HA mutations demonstrated in vitro has not yet been directly correlated with increased drug resistance in humans. Reduced NAI susceptibility associated with HA mutations in cell culture may not necessarily translate to reduced susceptibility in vivo because of differences in sialyl acid distribution patterns and HA binding requirements [5, 37]. It is often difficult to define the selective pressure that leads to the emergence of HA substitutions that circulate in humans or emerge in NAI-treated patients. Thus, many HA mutations are pleiotropic in that they may be selected by NAIs, anti-HA antibodies, or changes in sialyl receptor distributions in a new host.

The clinical relevance of the studied HA mutations on susceptibility to NAIs remains unknown and they may represent cell culture and host adaptations. Several mutations did emerge during clinical use of NAIs or have been observed in clinical specimens. For example, the HA1 S162 N mutation was selected during intravenous zanamivir treatment [23]. Subjects with the HA1 D222G mutation had significantly longer ICU stays: 22.8 days vs 14.0 days for those without this substitution. The D222G substitution was also found with considerable frequency in fatal and severe cases but was virtually absent among clinically mild cases [18, 40]. Consistent with the previous reports, our data showed that recombinant virus containing D222G resulted in ~ 2 log_10_PFU/mL higher titers over 72 h of replication in cell culture.

We found that several of the HA mutations associated with reduced susceptibility to NAIs correlate with decreased immune reactivity to polyclonal goat or ferret anti-influenza CA/04 antisera as measured by HI assay. Viruses containing two specific HA mutations (G155E and D222G) also demonstrated significantly decreased inhibition by anti-CA/04 mAbs and human sera collected from three different donors in MN assay. It is worth noting that these HA mutations were shown to be associated with antigenic drift in previously circulating H1N1 viruses and were also selected in escape mutants under mAb selective pressure [17, 31, 39, 44]. Our findings suggest that chronic use of NAIs such as oseltamivir, zanamivir, or peramivir may promote acquisition of HA mutations that correlate with reduced sensitivity to inhibition by anti-influenza antibodies. The potential exists that NAIs selective pressure can contribute to the HA antigenic evolution.

## Conclusions

Vaccination and use of anti-influenza drugs are two major approaches to the prevention and treatment of influenza virus infection. However, the activity of vaccines and antivirals can be altered by adaptive changes in the HA protein. Our findings indicate that exposure to NAIs may be associated with acquisition of HA mutations that correlate with escape from natural or vaccine-induced immunity to influenza virus.

## Data Availability

The datasets used and analyzed during the current study are included within this article.

## Abbreviations

ANOVA: analysis of variance
CA/04: A/California/04/09 A(H1N1)pdm09 influenza virus
HA: hemagglutinin
HI: hemagglutination inhibition (HI)
mAbs: monoclonal antibodies
MDCK: Madin-Darby canine kidney cells
MN: microneutralization
NA: neuraminidase
NAIs: neuraminidase inhibitors,
PFU: plaque-forming unit
RBS: receptor binding site
TCID_50_: 50% tissue culture infectious dose
